# Segmentation of patients with small cell lung cancer into responders and non-responders using the optimal cross-validation technique

**DOI:** 10.1186/s12874-024-02185-7

**Published:** 2024-04-08

**Authors:** Elham Majd, Li Xing, Xuekui Zhang

**Affiliations:** 1 Department of Mathematics and Statistics, University of Victoria, Victoria, BC Canada; 2 Department of Mathematics and Statistics, University of Saskatchewan, Saskatoon, SK Canada

**Keywords:** Best overall response, Clinical trials, Cross-validation, Overall survival

## Abstract

**Background:**

The timing of treating cancer patients is an essential factor in the efficacy of treatment. So, patients who will not respond to current therapy should receive a different treatment as early as possible. Machine learning models can be built to classify responders and nonresponders. Such classification models predict the probability of a patient being a responder. Most methods use a probability threshold of 0.5 to convert the probabilities into binary group membership. However, the cutoff of 0.5 is not always the optimal choice.

**Methods:**

In this study, we propose a novel data-driven approach to select a better cutoff value based on the optimal cross-validation technique. To illustrate our novel method, we applied it to three clinical trial datasets of small-cell lung cancer patients. We used two different datasets to build a scoring system to segment patients. Then the models were applied to segment patients into the test data.

**Results:**

We found that, in test data, the predicted responders and non-responders had significantly different long-term survival outcomes. Our proposed novel method segments patients better than the standard approach using a cutoff of 0.5. Comparing clinical outcomes of responders versus non-responders, our novel method had a p-value of 0.009 with a hazard ratio of 0.668 for grouping patients using the Cox proportion hazard model and a p-value of 0.011 using the accelerated failure time model which approved a significant difference between responders and non-responders. In contrast, the standard approach had a p-value of 0.194 with a hazard ratio of 0.823 using the Cox proportion hazard model and a p-value of 0.240 using the accelerated failure time model indicating the responders and non-responders do not differ significantly in survival.

**Conclusion:**

In summary, our novel prediction method can successfully segment new patients into responders and non-responders. Clinicians can use our prediction to decide if a patient should receive a different treatment or stay with the current treatment.

**Supplementary Information:**

The online version contains supplementary material available at 10.1186/s12874-024-02185-7.

## Background

Small Cell Lung Cancer (SCLC) represents $15\%$ of all lung cancers and is known for its highly invasive capacity and early metastatic behavior [1]. Treatments for SCLC have changed very little in the past 20 years compared to other types of lung cancer [2]. Nearly two-thirds of patients with SCLC have an extensive-stage disease at diagnosis, which is associated with poor prognosis and a 5-year survival rate of $7\%$ [3, 4]. Different treatment methods are used to treat almost all patients with advanced SCLC; however, identifying robust predictive biomarkers remains challenging. Unsatisfactory predictive accuracy has restricted real-world clinical practice [5].

In oncology, several clinical endpoints have been considered for assessing treatment efficacy [6, 7]. The primary endpoint is investigator-assessed Progression-Free Survival (PFS) measured from the date of randomization to the date of objective disease progression or death from any cause, whichever has occurred earlier. The secondary endpoint is Overall Survival (OS). OS is defined as the time from randomization to the date of death (any cause) or to the date of last patient contact (censoring date). Indeed, time-to-event outcomes are used in medical research since they offer more information than simply whether an event occurred. To handle these outcomes, as well as censored observations in which the event is not observed during follow-up, survival analysis methods should be used [8]. OS is recognized as the gold standard for assessing treatment efficacy in the Randomized Controlled Trials (RCTs) of anticancer therapies [9–11]. OS as the primary endpoint can be challenging because it necessitates a huge sample size, long follow-up times, and a growing line of therapies that may detect the results [12]. Other secondary endpoints are Overall Response Rate (ORR), percentage of patients with Complete Response (CR), or Partial Response (PR) [13]. According to the previous study, the BOR was defined as a record of the best outcomes from the beginning of the study to the end of the treatment [5]. Considering the cost and duration required for clinical trials, segmenting patients into responders and non-responders at the early stages is vital. A probability threshold is often used to segment patients through machine learning methods. Besides, predictive model validation strategies are common in prediction, which includes (repeated) train/test data splits or re-sampling techniques such as CV [14]. Several aspects include the selection of variables, size of datasets, imbalanced data, and Cross-Validation (CV) technique, which can impact may impact the performance of predictions.

It was a commonly accepted assumption that the measured performance of the predictive models using the validation set was an unbiased estimator of the performance of such models in general. However, multiple recent studies have revealed that this assumption does not always hold [15]. Westerhuis et al. [16] confirmed that the performance measured by CV can be over-optimal. Harrington et al. [17] proved that a single split between the training and test set may provide an erroneous estimation of model performance.

In addition, the results of previous studies show that, to have a stable estimation of model performance, a good balance between the training and the test set is essential. There is no clear evidence to suggest which CV technique would give the best results [15]. Several studies were carried out [18, 19] to predict OS derived from tumor growth dynamics and to consider a probability threshold of 0.5 to segment patients. In these studies, researchers did not focus on the method of CV that works on a specific clinical trial dataset. Indeed, many researchers did not notice the impact of an optimal CV technique on the accuracy of prediction results for finding a suitable cutoff value to segment the patients. Several studies applied the model based on one common CV technique used in similar studies.

Chang et al. 2022 [20] evaluated disease prognosis among patients with diffuse large B-cell lymphoma using machine learning models with an iterated CV method [20]. In this study, 5-folds with 10-iterated were conducted, which resulted in 50 testing results. This study did not mention the reason for selecting 5-folds with a 10-iterated technique. In another study, [5], the SVM classification of somatic mutations based on 5-folds was applied to predict the BOR in patients with EGFR/ALK-negative NSCLC treated with anti-PD-1. This study did not mention why the 5-fold CV was the best technique for their predictive model.

Overall, the gap exists in previous research to segment the patients into responders and non-responders with the best cutoff value to group patients. The popular method uses a probability threshold of 0.5 to convert the probabilities into binary group membership. It is vital to get a more accurate cutoff that can be used for the segmentation of patients. Therefore, the motivation to undertake this study is to propose machine-learning methods for clinical trials to segment patients according to the best cutoff through the optimal CV technique.

## Methods

This section describes the features of building the machine learning methods, details about the datasets, model diagram, and data analysis pipeline in this study.

### Features

The features used to build the model were baseline characteristics, tumor assessments in the early stages, the BOR, and OS. The predictor variables comprised baseline weight, age, sex, race, and smoking status of each patient. Besides, the percentage of tumor size changes from baseline tumor size in the longest diameters was computed for the fourth visit as the landmark time point. The reason for considering the value of features until the fourth visit was to propose the model for the segmentation of the patients in the early stages of treatment.

The BOR and OS of each patient were used in building the predictive models and segmenting the patients into responders and non-responders. The BOR, a response variable, was considered as the categories of PR and CR vs. SD and PD.

### Datasets

**Fig. 1:**
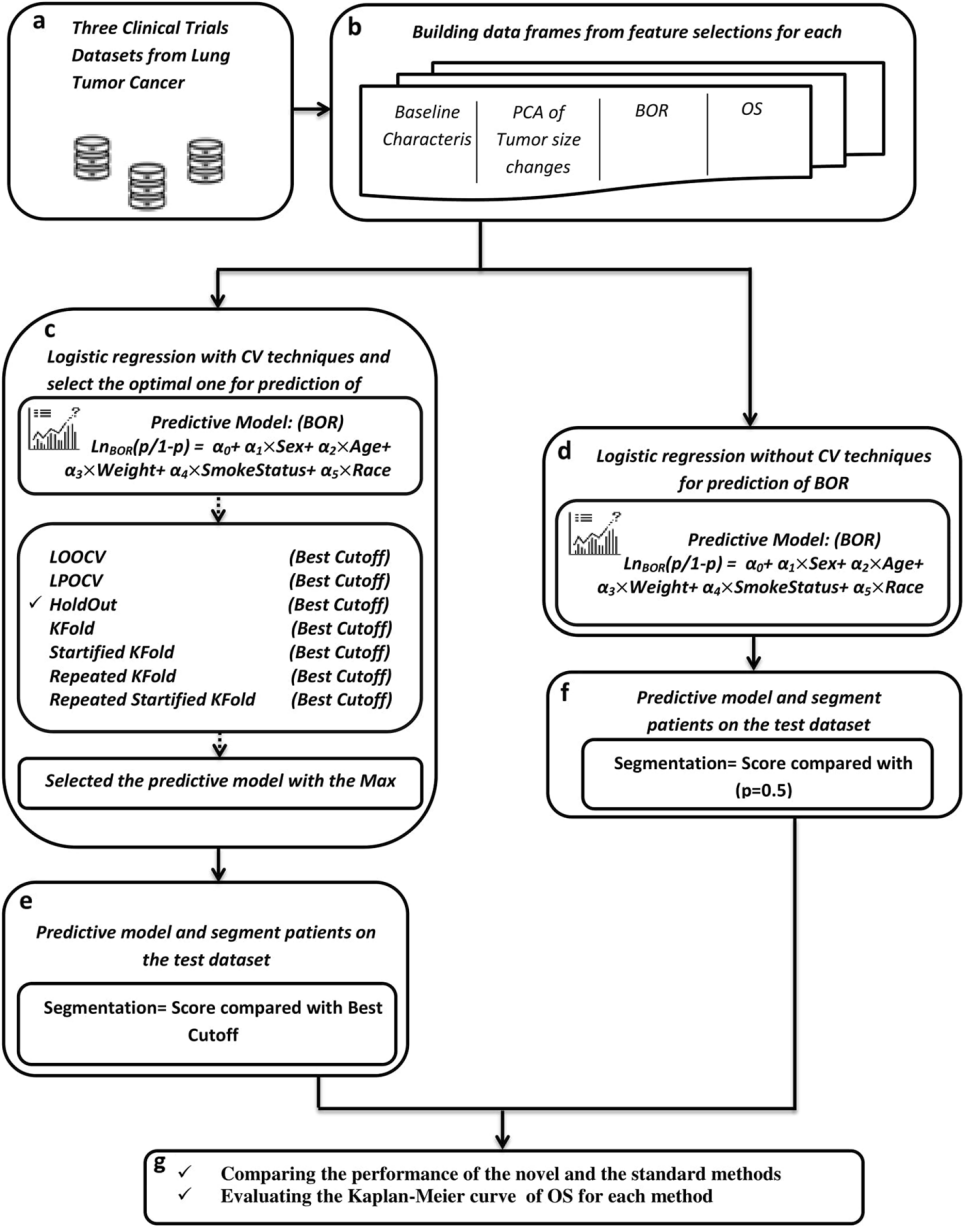
Model diagram schema. The process of the model diagram is shown in a different section. **a** presents the selection of the datasets from the Project Data Sphere’s Data Sharing Platform that contained all features for building the predictive models. **b** shows the data frames made by features in training datasets and then made a model to predict the BOR. **c** shows the method used to predict the BOR. In this method, two training datasets were considered, and test data was used to segment the patients. The novel method applied seven main CV techniques to explore the optimal CV techniques. **d** is the standard method that built the predictive models and used two training datasets. **e** identifies that in the standard method the best cutoff was collected from the optimal CV techniques applied to segment the patients. **f** shows, in the standard method, a probability threshold of 0.5 converted the score of patients into binary group membership. Finally, **g** shows the comparison of the performances among predictive models using the CPH and AFT models, in addition to, presenting the Kaplan-Meier Curve for each method

The clinical trial databases were derived from the Project Data Sphere’s Data Sharing Platform (Fig. 1a). Project Data Sphere has successfully restored many datasets on cancer patients and delivered community access to these data [21, 22].

This study contained three datasets: small cell lung cancer with the baseline characteristics, tumor assessment information, BOR, and OS. The number of patients was different in each dataset. The details of the datasets are presented in Table 1.

**Table 1:** Details of datasets. The numbers of females and males, the range of patients’ ages, the smoking status of patients, and the category of weights and race among patients for each dataset are summarized separately

Dataset	NCT02499770	NCT02514447	NCT03041311
Total Numbers	962	146	670
Female	280	69	278
Male	682	77	392
18-$\ <65$	479	100	390
65-75	391	34	236
$\ >75$	92	12	44
Never Smoked	0	11	53
Former Smoker	654	75	317
Current Smoker	962	60	300
Weight$\ <=75$	619	64	266
Weight$\ >75$	343	82	404
Caucasian	825	145	455
Non-caucasian	172	7	23

### Model diagram

This study contained a novel and a standard method, as shown in Fig. 1. In the novel method, the predictive models were built using two training datasets for computing the best cutoff using CV techniques. The outcomes of two training datasets resulted in the prediction of BOR and got the best cutoff. Differences between segmented patients were tested on the third dataset to investigate the model’s performance. For every training dataset, a data frame was made from the features (Fig. 1b), including the baseline characteristics. The subset of each data frame made by Principal Component Analysis (PCA) of percentage tumor size changes from baseline tumor size for four early visits.

After preprocessing the data frames, the predictive models were built to predict BOR using logistic regression and seven CV techniques: LOOCV, LPOCV, HoldOut, K-fold, Stratified K-fold, Repeated K-fold, and Repeated Stratified K-fold (Fig. 1c). Due to the fact that accuracy is not the best choice for detecting the best cutoff, the F1-score was considered getting the best cutoff. The predictive models with the maximum cutoff value were selected. Then the coefficient of selected models’ features was used as scores to make the scoring system on the test dataset for segmentation of the patients into the responders and non-responders (Fig. 1e).

The standard Method was a common method in clinical trials to segment the patients using a probability threshold of 0.5 to convert the probabilities into binary group membership. In this method, the logistic regression model was applied with no CV techniques. The coefficients of the features collected from the training set were used for the test dataset.

The Kaplan-Meier curves, to present the segmentation of the patients, were applied for two methods using the best cutoff values in the novel method and the score of patients and the probability threshold of 0.5 for the standard model (Fig. 1f). The Kaplan-Meier method establishes survival curves, which is the basic statistical method of analysis. It is a non-parametric method in that no mathematical form of the survival distributions is assumed [8]. A p-value less than 0.05 is considered significant [23]. Besides, to assess the performance of the two methods, Cox proportional hazards (CPH) model and the Accelerated Failure Time (AFT) model were evaluated for each method. The CPH model is known as the standard method to study the relationship between covariates and survival [24]. Although most cancer researchers apply the proportional hazard model, the AFT model in comparable conditions - as they do not need proportional hazards assumption and analyze a parametric statistical distribution for survival time - will be a credible option [25]. Thus, in this study, CPH and AFT were used to compare the performance of the novel and standard methods. Finally, to evaluate their respective goodness-of-fit the Akaike Information Criterion (AIC) was used to find which method had a lower AIC which defines a better fit.

### Data analysis pipeline

The predictive models were produced from a training dataset. First, the data frame was created of variables, including Subject ID, Sex, Age, Weight, Smoking Status, and Race for each patient (Fig. 1b). Then a subset of the data frame was made using tumor assessment for patients with four early visits. The percentage of tumor size changes was computed using the baseline tumor size. Then the PCA was computed on the percentage change in tumor size. For this purpose, the collapse data was constructed across visits to make tumor assessment levels. Then the PCA was calculated for the fourth early visit. A combination of baseline characteristics and Principal Component Scores (Pcs) led to building the predictive models for the prediction of BOR.

In the novel method (Fig. 1c), the designed predictive models predict the BOR based on seven CV techniques and two training datasets. The seven main CV techniques are:

#### Leave-one-out cross-validation

One of the CV techniques used in this study was Leave-one-out cross-validation (LOOCV). LOOCV is a special case of k-fold CV, in which the number of folds equals the number of instances. When the number of instances either in a data set or for a class value is small, such as gene microarray data and gene sequence data, LOOCV should be adopted to get a reliable accuracy estimate for a classification algorithm [26].

#### Leave-pair-out cross-validation

Another CV technique was Leave-pair-Out Cross-validation (LpOCV) with $p=2$ observations. LpOCV applies *p* observations as the validation set and the remaining observations as the training set. It is repeated in all ways to cut the original sample of a validation set of p observations and a training set [27]. A variant of LpOCV with $p=2$ has been suggested as a nearly unbiased method for estimating the area under the ROC curve of binary classifiers [28]. Smith et al. 2014 [29] demonstrated that sample splitting, CV without replication, and Leave-One-Out Cross-Validation (LOOCV) produced optimism-adjusted estimates of the concordance statistic that might be associated with greater absolute error than other available CV techniques [29].

#### HoldOut

To split the data into approximately $80\%$ training set and about $20\%$ testing set, the HoldOut CV technique was used. In the holdout method, the dataset should randomly assign data points to two sets (train and test). The HoldOut approach is often applied when an external validation dataset is not available. However, this approach does not lead to truly external validation [30]. This study applied the HoldOut CV technique to split data into $75\%$ training and $2\%$ testing.

#### K-folds

In CV techniques, the model is assessed in all the subjects. However, each model always contains a smaller sample size than the total, typically $90\%$ (i.e., in the case of 10-fold CV); hence, there remains more uncertainty in the coefficients than if the whole data set had been used. Thus, the CV technique might be most effective when the number of subjects eliminated when generating a given predictive model is the lowest [30]. This study considered K-fold CV with K between 3 and 10. The proposed novel methods explored which K-fold performed better than others.

#### Repeated K-folds

Repeated CV helps to estimate the mean of all possible K-fold CV over the given data [31]. Several studies were carried out to find out whether K-fold should be repeatedly performed to get reliable accuracy estimates. Vanwinckelen and Blockeel [31] showed that repeated CV should not be assumed to give much more precise estimates of a model’s predictive accuracy [31]. In this study, repeated K-fold was used with *K* between 3 and 10 and repeated between 2 and 4.

#### Stratified K-folds

Stratified is the extended form of a CV technique. In Stratified K-fold, the distribution of a class is done among *n* number of folds. The distribution of a class in each fold of the dataset is the same as present in the original dataset. Regular CV arbitrarily partitions *S* into *n* folds without taking class distributions into account. K-Fold CV could cause a certain class to be distributed unevenly, with some folds containing more cases of the class than others [32]. This study applied the Stratified K-fold technique with *n* between 3 and 5.

#### Repeated stratified K-folds

This technique is like the stratified k-folds CV, but it is repeated *n* times [33]. Thus, the stratified k-fold process is repeated $k\times n$ times [34]. In this study, repeated Stratified K-fold was used with splits between 3 and 5 and repeated between 2 and 4.

As practically comparing the computational time between different CV techniques, LOOCV is computationally very expensive. LOOCV needs less computation time than LpOCV because there are only $\ C_1^n=n$ passes rather than $\ C_p^n$. However, *n* passes can still require quite a large computation time [35].

In addition, the Holdout is the simplest CV technique, while the data set is divided into *K* subsets and the HoldOut method is repeated *K* times in the K-fold CV technique. Thus, the computational time of HoldOut is mostly less than K-fold. Although increasing the number of *K* causes rising in computational time [26].

In the novel method, the CV technique with the maximum best cutoff values was chosen as the optimal CV technique. In contrast, the standard method computes the prediction of BOR without CV techniques. After storing the prediction results from methods one and two, the coefficients for each feature of the selected CV techniques in each method were used as a score of each feature for the test dataset. The mean coefficients of each feature were collected from two training datasets in the novel method. Then the scoring system was built to get the score of the test dataset to segment the patients using the best cutoff collected from the optimal CV techniques. The standard method did not use the CV technique and a probability of 0.5 converted probabilities into responders and non-responders. The scoring system for each method was calculated separately:

1 $$\begin{aligned} Score_{mtp}=\left(\sum \limits _{i=d}\gamma _{mip}\right)/2\times (Sex_{mdp})+\left(\sum \limits _{i=d}\sigma _{mip}\right)/2\times (Age_{mdp})+ \nonumber \\ \left(\sum \limits _{i=1,2}\lambda _{mip}\right)/2\times (Weight_{mdp}) +\left(\sum \limits _{i=1,2}\delta _{mip}\right)/2\times (Smoke_{mdp})+ \nonumber \\ \left(\sum \limits _{i=1,s}\phi _{mip}\right)/2\times (Race_{mdp})+\left(\sum \limits _{i=1,s}\mu _{mip}\right)/2\times (Pcs_{mdp})+\left(\sum \limits _{i=1,s}\eta _{mip}\right)/2 \end{aligned}$$

Where $Score_{mtp}$ shows the scoring system of method, *m*, for test dataset, *t*, per patient, *p*. The value of, *m*, is the count of methods which can be one, which means novel method, or two, which means the standard method. *i* shows the count of training datasets that are between one and two. $(\sum _{i=d}\gamma _{mip})/2$ is the mean coefficients for the sex feature of the method, *m*, and training dataset, *d*, per patient, *p*. $Sex_{mdp}$ is the sex of each patient, *p*, for method, *m*, and training dataset, *d*. $\ (\sum _{i=d}\sigma _{mip})/2$ is the mean of coefficients for the age feature of the method, $\ m$, for the selected training dataset, *d*. $Age_{mdp}$ shows the age of each patient, *p*, for method, $\ {m}$, and training dataset, *d*, per patient, *p*. $\ (\sum _{i=d}\lambda _{mip})/2$ is the mean coefficients for the weight feature of the method, $\ {m}$, for the training dataset, *d*. $Weight_{mdp}$ represents the weight of each patient, *p*, for method, $\ {m}$, and training dataset, *d*, per patient, *p*. $(\sum _{i=d}\delta _{mip})/2$ is the mean coefficients for the smoking status feature of the method, *m*, for the training dataset, *d*, per patient *p*. $Smoke_{mdp}$ is the smoking status of each patient, *p*, for method, *m*, and training dataset, *d*. $\ (\sum _{i=d}\phi _{mip})/2$ is the mean coefficients for the race feature of the method, *m*, for the training dataset, *d*, per patient, *p*. $Race_{mdp}$ is the race of each patient, *p*, for method, $\ {m}$, and selected training dataset, *d*. $(\sum _{i=d}\mu _{mip})/2$ shows the mean coefficients for the Principal Components (PCs) feature of the method, *m*, for the training dataset, *d*, per patient *p*. $\ Pcs_{mdp}$ is the PCs value of method, $\ {m}$, for training dataset, *d*, per patient, *p*. $(\sum _{i=d}\eta _{mip})/2$ reveals the mean intercepts of method, *m*, and training dataset, $\ {d}$, per patient *p*.

Then, to group patients in the novel method, the probability of each patient computed by the scoring system, $P_{Score_{mtp}}$, was compared with the mean best cutoff value got from the models with the optimal CV technique:

2 $$\begin{aligned} Group_{mtp}=P_{Score_{mtp}}>Bcut_{mt}/2 \end{aligned}$$

Where $Group_{mtp}$ shows the segmentation of method, *m* and test dataset, *t*, per patient, *p*. $P_{Score_{mtp}}$, represents the score of each patient *p* comes from the scoring system, $Score_{mtp}$, for method, *m*, in every test dataset, *t*, per patient, *p*, and, $Bcut_{mt}$ is best cutoff value for method, *m* and test dataset, *t* comes from the mean of best cutoff values of two training datasets.

### Standard method

The standard method used logistic regression to build the predictive model with the same features as the novel model, but in this method, the prediction was carried out with no CV method and two training datasets. The mean coefficients of each feature were used in the test dataset to get the score. Then the score of patients was calculated through the prediction results from the training dataset on the test datasets, as described in Eq. 1. The patients were segmented into responders and non-responders using the cutoff 0.5. Then, Kaplan-Meier curves summarized time-to-event endpoints, estimated median times with $95\%$ CIs, and revealed the segmentation by using the $Group_{mtp}$ collected from each method. The CPH and the AFT models were also applied to assess the performance of the novel and standard methods.

## Results

This section describes the outcomes of each method with details on the datasets. Besides, the segmentation results of patients into responders and non-responders between the novel and standard methods. The basic characteristics of the patients in each dataset after prepossessing the data are shown in Table 1. Most patients are Male, and the age of patients between 18 and 64 years is higher than other ages. In addition, the numbers of current smokers are more.

As described in the model diagram section, in the novel method, the best cutoffs from the performances of the predictive models regarding the optimal CV techniques were used to segment the patients. The optimal CV technique was selected according to the performances of the predictive model for each dataset in the novel method. The optimal CV technique for dataset (NCT02499770) was 2 Repeated-10 Fold with the best cutoff value of 0.722, while in the (NCT03041311), the optimal CV technique was 4 Repeated-7 Fold with the best cutoff value of 0.880, and the optimal CV technique for the third dataset (NCT02514447) was 2 Repeated-6 Fold. The results approved that there is no specific CV technique for all clinical trial datasets. The selected CV technique for each dataset in the novel method was different, while all datasets had similar features, data types, pre-processing, and predictive models. Although the size of the datasets was different and the proportion of data in each dataset was various, as shown in Table 1. These results confirmed that the specific CV technique could not apply to all clinical trial datasets. In that case, the coefficient of features and the best cutoff value as a threshold to segment the patients can be affected. Using one CV technique for no specific reasons may hurt the prediction results and the segmentation of patients.

**Fig. 2:**
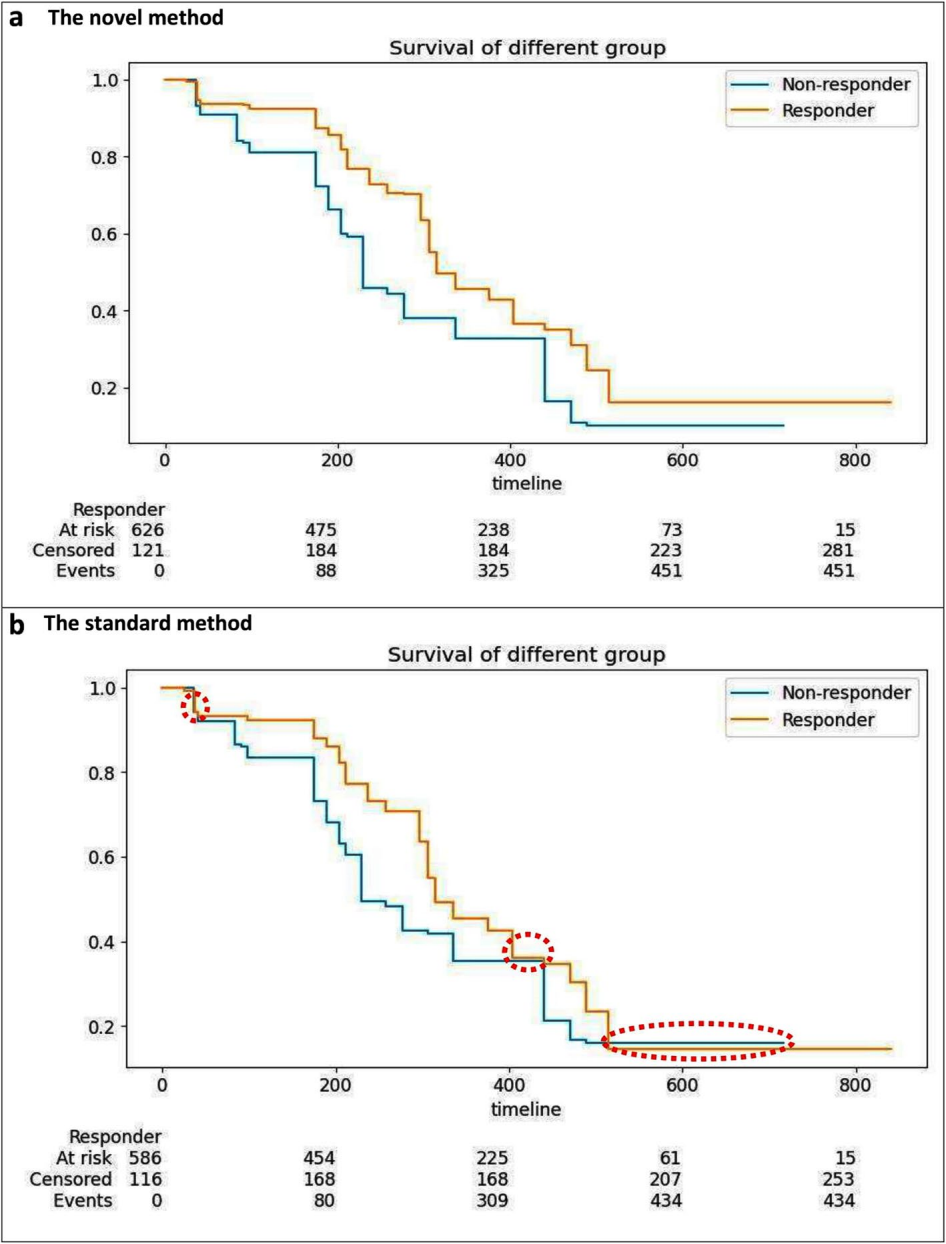
The Kaplan-Meier Curve of the novel and the standard methods. **a** presents the Kaplan-Meier Curve for the novel method with significant segmentation. **b** shows the Kaplan-Meier Curve with the standard method. The highlighted red hatched circles reveal the unacceptable performance of the standard method in the segmentation of responders and non-responders

The performance of predictive models for the novel and the standard methods are summarized in Table 2. As shown in Table 2, the accuracy of predictions for the novel method is over 0.7 and higher than the standard method. Besides, the Mean Squared Error (MSE) of the novel method was less than the standard method among all datasets. It means that the selected predictive models with optimal CV techniques had a better performance than the standard method. The results for the segmentation of patients were analyzed by using Kaplan-Meier curves. Figure 2 presents the Kaplan-Meier curves for the test dataset (NCT0249970) using the novel and the standard methods. As shown in Fig. 2, the Kaplan-Meier curves for the novel method could significantly separate responders and non-responders, whereas the standard model’s Kaplan-Meier curves had inseparable portions in three regions that are highlighted with red hatched circles. Such a visual pattern indicates the best cutoff obtained from the optimal CVs can segment the patients more accurately than the standard cutoff.

**Table 2:** The value of prediction features, including Accuracy, MSE, Specificity, Precision, and Recall were summarized for the novel method using the best cutoff and standard method using the probability threshold 0.5 to segment the patients

Method	Dataset	Accuracy	MSE	Specificity	Persicion	Recall
Novel	NCT02499770	0.717	0.283	0.815	0.729	0.717
Novel	NCT02514447	0.100	0.000	0.100	0.100	0.100
Novel	NCT02499770	0.688	0.312	0.715	0.680	0.688
Standard	NCT02499770	0.688	0.312	0.715	0.680	0.688
Standard	NCT02514447	0.910	0.090	0.886	0.910	0.910
Standard	NCT03041311	0.849	0.151	0.877	0.847	0.849

Besides, the clear visual patterns observed in Fig. 2, numeric results from test and regression results also indicate novel method works better than the standard cutoff. The CPH model was fitted using the test dataset (NCT0249970) to compare the patient survival outcomes between the two segmented groups. In the regression, the covariates sex, age, weight, race PCs, and the group were adjusted. Comparing the two patient groups segmented by the novel method has a *p*-value of 0.099 and a hazard ratio of 0.668 with $95\%$ confidence interval $[-0.707, -0.101]$, which indicates the two groups have significantly different clinical outcomes after adjusting to important covariates. In contrast, the two groups, segmented by the standard approach, have a *p*-value of 0.194 which is over 0, 05 with a hazard ratio of 0.823 with $95\%$ confidence interval $[-0.489, 0.099]$. The details of Cox regression results are shown in Table 3. As a sensitivity analysis, the AFT model was also fitted as an alternative to Cox regression and got similar results with the p-value of 0.011 for the novel method, whereas the p-value of 0.240 for the standard method, as shown in Table 4. Besides, the AIC value of the novel method, 7947.917, was less than the standard method, 7952.950, which means the model had a better fit in the proposed novel model than the standard approach.

**Table 3:** Cox proportional hazard for the novel and standard method with test dataset (NCT02499770)

Novel Method	HR	exp(c) lower$95\%$	exp(c) upper$95\%$	*p*
Covariate				
PC2	1.211	0.975	1.504	0.083
Sex	0.271	0.183	0.401	<0.0005
Age	1.579	1.147	2.175	0.005
Smoke	4322.988	25.543	7.316e+05	0.001
Weight	0.989	0.971	1.007	0.230
Race	0.172	0.040	0.736	0.018
Group	0.668	0.494	0.903	0.009
Standard Method	HR	exp(c) lower$95\%$	exp(c) upper$95\%$	*p*
Covariate				
PC2	19.453	0.040	9400.350	0.347
Sex	9.787	5.172	18.520	<0.0005
Age	1.251	1.023	1.530	0.029
Smoke	2.219	1.410	3.491	0.001
Weight	1.036	0.990	1.085	0.126
Race	3.135	1.484	6.622	0.003
Group	0.823	0.613	1.104	0.194

**Table 4:** Accelerated failure time model for the novel and standard method with test dataset (NCT02499770)

Novel Method	HR	exp(c) lower$95\%$	exp(c) upper$95\%$	*p*
Covariate				
Age	0.849	0.708	1.019	0.078
PC2	0.906	0.801	1.026	0.120
Race	5.752	2.559	12.931	<0.0005
Sex	1.910	1.531	2.382	<0.0005
Smoke	0.028	0.002	0.509	0.016
Weight	1.011	1.001	1.021	0.031
Group	1.254	1.053	1.493	0.011
Standard Method	HR	exp(c) lower$95\%$	exp(c) upper$95\%$	*p*
Covariate				
Age	0.936	0.835	1.049	0.255
PC2	0.266	0.008	9.025	0.461
Race	0.352	0.233	0.533	<0.0005
Sex	0.316	0.221	0.451	<0.0005
Smoke	0.706	0.548	0.910	0.007
Weight	0.969	0.945	0.993	0.011
Group	1.107	0.934	1.311	0.240

We repeat this analysis on another clinical trial, NCT00981058, an open-label Phase 3 study designed to investigate the overall survival (OS) of patients diagnosed with Stage IV squamous non-small cell lung cancer (NSCLC). Analysis results of this data also demonstrate that the proposed approach outperforms the standard method in all performance metrics (including MSE, accuracy, precision, and recall) for patient segmentation. More details are given in the Supplementary document.

## Discussion

This study used machine learning methods to segment patients into two groups: responders and non-responders in the early stages. A novel data-driven approach for selecting a better cutoff value based on the optimal CV technique was proposed in this study. Two methods were applied to segment the patients. The novel method used the seven CV techniques to predict BOR with two training datasets. The mean coefficients of features and the best cutoff value were collected from the optimal CV technique for each dataset used to group the patients into responders and non-responders. In the standard method, the training dataset was applied to predict BOR, and the results were used to segment the patients into test data with a probability threshold of 0.5 to convert the probabilities into binary group membership. The results show although the datasets may have similar pre-processing, the same features and data types with different sizes of data. The optimal CV technique for predicting the BOR and the best cutoff value is different for each dataset. The novel method using the scoring system can successfully segment new patients, and the predicted responders and non-responders have significantly different long-term survival outcomes. Besides, results revealed that the best CV technique is uncertain in practice. Using the optimal CV technique to select a better cutoff value can result in significant segmentation of patients in the early stages compared to the standard model with the probability threshold of 0.5.

Another problem, also called ‘optimal cutoff selection’, often conflated with the problem discussed in this work, yet it significantly diverges in its goals and methods. This alternate problem seeks to determine the best cutoff to dichotomize a continuous variable, such as gene expression levels, for evaluating its link to an outcome like patient survival. The ’optimal’ here is geared towards enhancing the relationship between the derived binary variable and the outcome. It requires individual hypothesis testing for each cutoff, requiring multiple testing adjustments to control the Type I error rate. These tests are not independent, hence innovative methods can significantly enhance statistical power beyond what is achievable with traditional methods like the Bonferroni correction. So, methods developed for problem B focus on improve statistical power while control the false positive rates in multiple testing settings. For example, xTile adopt Monte Carlo or validation set methods to adjust inflated p-values due to multiple testing [36]. Permutation tests can be applied to establish a null distribution for order statistics to correct p-values for multiple testing [37], and this method has been integrated into web applications for prognostic biomarker identification in cancer research [38]. However, due to their computational intensity, permutation methods are often impractical for web applications, prompting the development of faster alternatives based on theoretical derivations of null distributions for order statistics [39]. Based on description above, we summarize the major differences between this problem and ours. This problem uses cutoff to dichotomize a predictor and needs to test its association with the outcome. In contrast, our problem dichotomizes predicted outcomes to make a decision. Method development for this problem focuses on multiple testing corrections, and our problem focuses on prediction performance.

For binary classification problems, the $P(y_i=1)$ probability space is divided into two segments, each representing one class ($y_i=1$ or $y_i=0$). Extending this to a multi-class problem means dividing a $K-1$ dimensional space into *K* regions, which could take shapes far more intricate (due to inter-threshold interactions) than simple threshold values can delineate. Furthermore, cross-validation to select multiple thresholds needs searching in high-dimensional space, which could substantially increase computational demands. In short, while cross-validation is effective for binary classification, it is not readily adaptable to multi-class contexts. To address this, we suggest an alternative method that involves two distinct data sets, or two subsets of a single data set. The classification model is trained on the first set, then applied to the second set to calculate the predicted class probabilities for each sample. These probabilities are then used as predictors, with the actual class labels as outcomes, to construct another machine learning model within the second set. This two-tier modeling allows for creating decision regions of various forms, surpassing the rigidity of multi-threshold cross-validation in higher dimensions. This technique is conceptually analogous to multi-task prediction models based on stacking methods [40]. The primary caveat is that it requires a sufficiently large dataset to train the conversion model on the second data set effectively.

## Conclusions

In this study, the proposed novel data-driven approach segmented patients into responders and non-responders in the early stages of treatment. Using real clinical trial data, we demonstrate our novel method outperforms the standard approach. We also illustrate how to use train data to fit a model and apply it to segment patients in new clinical trials. The results show that our proposed model can accurately segment patients into responders and nonresponders that have significant differences in their clinical outcomes. Clinicians can use the proposed machine-learning method to decide if a patient should receive a different treatment or stay with the current treatment at the early stage.

### Supplementary Information

**Supplementary Material 1.**

## Data Availability

The source code used for data analysis is available at https://github.com/ubcxzhang/Tumor.Survival.CV. All datasets used in this project are downloaded from https://data.projectdatasphere.org/projectdatasphere/html/home.

## Abbreviations

AIC: Akaike Information Criterion
AFT: Accelerated Failure Time
BOR: Best Overall Response
CPH: Cox proportional hazards
CV: Cross-Validation
OS: Overall Survival
PR: Partial Response
CR: Complete Response
PD: Progressive Disease
SD: Stable Disease
ORR: Overall Response Rate
PFS: Progression-Free Survival
RCTs: Randomized Controlled Trials
NSCLC: Non- Small Cell Lung Cancer
SCLC: Small Cell Lung Cancer
SVM: Support Vector Machine
LOOCV: Leave-One-Out Cross-Validation
LpOCV: Leave-Pair-Out Cross-validation
MSE: Mean Squared Error
PCA: Principal Component Analysis
PCs: Principal Components
PFS: Progression-Free Survival

## References

[1] Zhou W, Wang P, Ti X, Yutian Y, Huang S, Yang Z (2022). Sequential Hypofractionated versus Concurrent Twice-Daily Radiotherapy for Limited-Stage Small-Cell Lung Cancer: A Propensity Score-Matched Analysis. Cancers..

[2] Keogh A, Finn S, Radonic T (2022). Emerging Biomarkers and the Changing Landscape of Small Cell Lung Cancer. Cancers..

[3] Liu SV, Reck M, Mansfield AS, Mok T, Scherpereel A, Reinmuth N (2021). Updated Overall Survival and PD-L1 Subgroup Analysis of Patients With Extensive-Stage Small-Cell Lung Cancer Treated With Atezolizumab, Carboplatin, and Etoposide (IMpower133). J Clin Oncol..

[4] Bernhardt EB, Jalal SI (2016). Small cell lung cancer. Lung Cancer..

[5] Peng J, Xiao L, Zou D, Han L (2022). A Somatic Mutation Signature Predicts the Best Overall Response to Anti-programmed Cell Death Protein-1 Treatment in Epidermal Growth Factor Receptor/Anaplastic Lymphoma Kinase-Negative Non-squamous Non-small Cell Lung Cancer. Front Med (Lausanne)..

[6] Mathoulin-Pelissier S, Gourgou-Bourgade S, Bonnetain F, Kramar A (2008). Survival end point reporting in randomized cancer clinical trials: a review of major journals. J Clin Oncol..

[7] Fleming TR, Powers JH (2012). Biomarkers and surrogate endpoints in clinical trials. Stat Med..

[8] George B, Seals S, Aban I (2014). Survival analysis and regression models. J Nucl Cardiol..

[9] Pazdur R (2008). Endpoints for assessing drug activity in clinical trials. Oncologist..

[10] Party W (2012). Guideline on the evaluation of anticancer medicinal products in man.

[11] Branchoux S, Sofeu CL, Gaudin AF, Kurt M, Moshyk A, Italiano A (2021). Time to next treatment or death as a candidate surrogate endpoint for overall survival in advanced melanoma patients treated with immune checkpoint inhibitors: an insight from the phase III CheckMate 067 trial. ESMO Open..

[12] Anagnostou V, Yarchoan M, Hansen AR, Wang H, Verde F, Sharon E (2017). Immuno-oncology trial endpoints: capturing clinically meaningful activity. Clin Cancer Res..

[13] Hamilton E, Cortes J, Ozyilkan O, Chen SC, Petrakova K, Manikhas A (2022). nextMONARCH Phase 2 randomized clinical trial: overall survival analysis of abemaciclib monotherapy or in combination with tamoxifen in patients with endocrine-refractory HR+, HER2-metastatic breast cancer. J Clin Oncol..

[14] Milá C, Mateu J, Pebesma E, Meyer H (2022). Nearest neighbour distance matching Leave-One-Out Cross-Validation for map validation. Methods Ecol Evol..

[15] Xu Y, Goodacre R (2018). On splitting training and validation set: A comparative study of cross-validation, bootstrap and systematic sampling for estimating the generalization performance of supervised learning. J Anal Test..

[16] Westerhuis JA, Hoefsloot HCJ, Smit S, Vis DJ, Smilde AK, van Velzen EJJ (2008). Assessment of PLSDA cross validation. Metabolomics..

[17] Harrington PdB (2018). Multiple versus single set validation of multivariate models to avoid mistakes. Crit Rev Anal Chem..

[18] Yu J, Wang N, Kågedal M. A new method to model and predict progression free survival based on tumor growth dynamics. CPT: Pharmacometrics Syst Pharmacol. 2020;9:177–84. 10.1002/psp4.12499 PMC7080535 32036626

[19] Claret L, Jin JY, Ferté C, Winter H, Girish S, Stroh M (2018). A model of overall survival predicts treatment outcomes with atezolizumab versus chemotherapy in non-small cell lung cancer based on early tumor kinetics. Clin Cancer Res..

[20] Chang CC, Chen CH, Hsieh JG, Jeng JH. Iterated cross validation method for prediction of survival in diffuse large B-cell lymphoma for small size dataset. Sci Rep. 2023;13(1):1438. 10.1038/s41598-023-28394-6. 10.1038/s41598-023-28394-6 PMC9876907 36697456

[21] Green AK, Reeder-Hayes KE, Corty RW, Basch E, Milowsky MI, Dusetzina SB (2015). The project data sphere initiative: accelerating cancer research by sharing data. Oncologist..

[22] Karpen SR, White JK, Mullin AP, O’Doherty I, Hudson LD, Romero K (2021). Effective data sharing as a conduit for advancing medical product development. Ther Innov Regul Sci..

[23] Badic B, Bouvier AM, Bouvier V, Morvan M, Jooste V, Alves A (2022). Predictors of Survival in Elderly Patients with Metastatic Colon Cancer: A Population-Based Cohort Study. Cancers..

[24] Rossello X, González-Del-Hoyo M. Survival analyses in cardiovascular research, part I: the essentials. Rev Esp Cardiol (Engl Ed). 2022;75:67–76. 10.1016/j.rec.2021.06.003 34215548

[25] Zare A, Hosseini M, Mahmoodi M, Mahmoodi K, Zeraati H, Naieni KH (2015). A Comparison between Accelerated Failure-time and Cox Proportional Hazard Models in Analyzing the Survival of Gastric Cancer Patients. Iran J Public Health..

[26] Wong TT (2015). Performance evaluation of classification algorithms by k-fold and leave-one-out cross validation. Pattern Recognit..

[27] Dell’aversana P (2019). Comparison of different Machine Learning algorithms for lithofacies classification from well logs. Boll Geofis Teor Appl..

[28] Kavzoglu T, Tonbul H. Tonbul, H. An experimental comparison of multi-resolution segmentation, SLIC and K-means clustering for object-based classification of VHR imagery. Clin Cancer Res. 2018;39:6020–36.

[29] Smith GCS, Seaman SR, Wood AM, Royston P, White IR (2014). Correcting for optimistic prediction in small data sets. Am J Epidemiol..

[30] Eertink JJ, Heymans MW, Zwezerijnen GJC, Zijlstra JM, de Vet HCW, Boellaard R (2022). External validation: a simulation study to compare cross-validation versus holdout or external testing to assess the performance of clinical prediction models using PET data from DLBCL patients. EJNMMI Res..

[31] Vanwinckelen G, Blockeel H. On estimating model accuracy with repeated cross-validation. In: Bernard DB, Bernard M, Michaël R, Willem W, editors. Proceedings of the 21st Belgian-Dutch conference on machine learning BeneLearn and PMLS: 2012-05-24; Ghent, Belgium; 2012. p. 39–44.

[32] Bhagat M, Bakariya B (2022). Implementation of Logistic Regression on Diabetic Dataset using Train-Test-Split, K-Fold and Stratified K-Fold Approach. Natl Acad Sci Lett..

[33] Pedregosa F, Varoquaux G, Gramfort A, Michel V, Thirion B, Grisel O (2011). Scikit-learn: machine learning in Python. J Mach Learn Res..

[34] Tougui I, Jilbab A, Mhamdi JE (2021). Impact of the choice of cross-validation techniques on the results of machine learning-based diagnostic applications. Healthc Inform Res..

[35] Molinaro MA, Simon R, R PM. Prediction error estimation: a comparison of resampling methods. Bioinformatics. 2005;21:3301–7. 10.1093/bioinformatics/bti499 15905277

[36] Camp RL, Dolled-Filhart M, Rimm DL. X-tile a new bio-informatics tool for biomarker assessment and outcome-based cut-point optimization. Clin Cancer Res. 2004 11;10(21):7252–9. 10.1158/1078-0432.CCR-04-0713. 10.1158/1078-0432.CCR-04-0713 15534099

[37] Hilsenbeck SG, Clark GM (1996). Practical p-value adjustment for optimally selected cutpoints. Stat Med..

[38] Cheng X, Liu Y, Wang J, Chen Y, Robertson AG, Zhang X, et al. cSurvival: a web resource for biomarker interactions in cancer outcomes and in cell lines. Brief Bioinform. 2022;23(3):bbac090. 10.1093/bib/bbac090. 10.1093/bib/bbac090 PMC9116376 35368077

[39] Lan L, Cheng X, Xing L, Zhang X. BOSS – Biomarker Optimal Segmentation System. 10.48550/arxiv.2305.09090.

[40] Xing L, Lesperance M, Zhang X. Simultaneous prediction of multiple outcomes using revised stacking algorithms. Bioinformatics. 2019 01;36(1):65–72. 10.1093/bioinformatics/btz531. 10.1093/bioinformatics/btz531 31263871

