# Supplementary material for: Segmentation of patients with small cell lung cancer into responders and non-responders using the optimal cross-validation technique
Source: BMC Med Res Methodol. 2024 Apr 8;24:83. doi: 10.1186/s12874-024-02185-7 (PMC11000309; doi:10.1186/s12874-024-02185-7)
Supplement: Supplementary file 1 — Supplementary Material 1. [file 12874_2024_2185_MOESM1_ESM.pdf]

Supplementary for - 'Segmentation of patients with small cell lung cancer into responders and non-responders using the optimal cross-validation technique'

Elham Majd[1], Li Xing[2], Xuekui Zhang[1*]

Methods

The clinical trial database used in this study was sourced from the Project Data Sphere's Data Sharing Platform. This dataset focused on non-small cell lung cancer and included baseline characteristics, tumor assessment information, BOR, and OS. The Table S1 presents detailed information about each dataset. The dataset originates from a randomized, multicenter, open-label Phase 3 study designed to investigate the overall survival (OS) of patients diagnosed with Stage IV squamous non-small cell lung cancer (NSCLC). The study compares the outcomes of individuals receiving necitumumab plus gemcitabine and cisplatin chemotherapy (GC+N Arm) with those receiving gemcitabine and cisplatin chemotherapy alone (GC Arm) in the first-line metastatic setting. In addition to OS, key secondary objectives encompass the assessment of progression-free survival (PFS), objective response rate (ORR), time to treatment failure (TTF) in each arm, and the safety profile of necitumumab when combined with gemcitabine and cisplatin chemotherapy. This dataset provides comprehensive insights into the treatment efficacy and safety considerations for Stage IV squamous NSCLC patients, contributing valuable information to inform clinical decision-making for this specific patient cohort.

The construction of the model closely followed the steps and methodology outlined in the paper. A comprehensive set of features, comprising baseline characteristics, early-stage tumor assessments, BOR (Best Overall Response), and OS (Overall Survival), was employed. Predictor variables involved baseline weight, age, sex, race, and smoking status for each patient. Furthermore, the percentage change in tumor size from baseline, particularly in the longest diameters, was calculated at the fourth visit, serving as a significant landmark

*Corresponding author: Xuekui Zhang, email: ubcxzhang@gmail.com
[1]Department of Mathematics and Statistics, University of Victoria, Victoria, BC, Canada
[2]Department of Mathematics and Statistics, University of Saskatchewan, Saskatoon, SK, Canada

time point. The decision to incorporate features up to the fourth visit was made to customize the model for patient segmentation during the early stages of treatment.

To build predictive models and categorize patients into responders and non-responders, both BOR and OS were incorporated. BOR, serving as a response variable, was categorized into PR (Partial Response) and CR (Complete Response) versus SD (Stable Disease) and PD (Progressive Disease). We introduce an innovative data-driven approach for determining an improved cutoff value through the optimal cross-validation technique. This approach was implemented on a novel dataset comprising non-small cell lung cancer patients. Utilizing the three datasets discussed in the paper, we developed a scoring system to effectively segment patients. Subsequently, these models were applied to segment patients within the test data.

Results

Table S1 shows dataset appears to be diverse, with a significant number of patients across different demographics. The majority of patients are current smokers, and the age distribution spans from below 65 to over 75. The dataset's gender distribution is imbalanced, with more males than females. Additionally, the weight distribution shows a considerable number of patients in both weight categories. In the novel approach, patient segmentation was conducted by employing optimal cross-validation techniques to determine the best cutoffs from the performance of predictive models. The optimal cross-validation technique was selected based on the model's performance for the dataset, specifically utilizing 3 Repeated-10 Fold, and the identified best cutoff value was 0.566. Similarly, the standard model employed the 3 Repeated-10 Fold cross-validation technique, with the best cutoff value determined as 0.548. The prediction features for the novel and standard methods are summarized in Table 2. The novel method exhibited consistent improvement in all metrics, with smaller MSE, higher accuracy, precision, and recall on the NCT00981058 dataset.

Table S3 and Table S4, shows survival analysis results of Cox proportional hazard and Accelerated Failure Time (AFT) models respectively. We found although both the novel method and the standard method show a significant association between segmented group and survival outcome, the novel method can provide a narrower confidence interval of the hazard ratio.

Table S1: Details of datasets. The numbers of females and males, the range of patients' ages, the smoking status of patients, and the category of weights and race among patients for each dataset are summarized separately.

Dataset	NCT00981058
Total Numbers	109940
Female	16606
Male	93335
18- $<$ 65	69342
65-75	38565
$>$ 75	2033
Never Smoked	3
Former Smoker	10332
Current Smoker	99605
Weight $<=$ 75	65921
Weight $>$ 75	44019

Table S2: The value of prediction features, including Accuracy, MSE, Specificity, Precision, and Recall were summarized for the novel method using the best cutoff and standard method using the probability threshold 0.5 to segment the patients.

Method	Dataset	Accuracy	MSE	Specificity	Persicion	Recall
Novel	NCT00981058	0.710	0.230	0.681	0.712	0.700
Standard	NCT00981058	0.690	0.310	0.610	0.692	0.690

Table S3: Cox proportional hazard for the novel and standard method with test dataset (NCT00981058).

Novel Method	HR	exp(c) lower95%	exp(c) upper95%	p
covariate				
PC2	0.997	0.997	0.998	0.011
Sex	0.998	1.000	1.000	<0.0005
Age	1.000	0.998	0.998	<0.0005
Smoke	1.000	0.988	1.000	<0.0005
Weight	1.000	1.000	1.000	<0.0005
Race	1.000	1.000	1.000	<0.0005
Group	1.177	1.164	1.210	<0.0005
Standard Method	HR	exp(c) lower95%	exp(c) upper95%	p
Covariate				
PC2	1.000	1.000	1.000	0.014
Sex	1.002	1.002	1.003	<0.0005
Age	1.000	1.000	1.000	<0.0005
Smoke	1.003	1.002	1.003	<0.0005
Weight	1.000	1.000	1.000	<0.0005
Race	1.000	1.000	1.000	<0.0005
Group	1.195	1.152	1.240	<0.0005

Table S4: Accelerated failure time model for the novel and standard method with test dataset (NCT00981058).

Novel Method	HR	exp(c) lower95%	exp(c) upper95%	p
Covariate				
Age	0.996	0.995	0.996	<0.0005
PC2	1.000	0.909	1.000	0.004
Race	1.001	1.000	1.001	<0.0005
Sex	0.985	0.985	0.995	<0.0005
Smoke	0.996	0.996	0.996	<0.0005
Weight	1.000	1.000	1.000	<0.0005
Group	0.755	0.727	0.798	<0.0005
Intercept	121.899	117.011	2153.546	<0.0005
Intercept	0.810	0.811	0.813	<0.0005
Standard Method	HR	exp(c) lower95%	exp(c) upper95%	p
Covariate				
Age	1.000	1.000	1.00	<0.0005
PC2	1.000	0.909	1.000	0.007
Race	1.001	1.00	1.001	<0.0005
Sex	0.996	0.996	0.997	<0.0005
Smoke	0.996	0.996	0.996	<0.0005
Weight	1.000	1.000	1.000	<0.0005
Group	0.770	0.736	0.805	<0.0005
Intercept	131.741	116.666	2148.764	<0.0005
Intercept	0.822	0.817	0.827	<0.0005
